# Diagnostic Value of Radiolabelled Somatostatin Analogues for Neuroendocrine Tumour Diagnosis: The Benefits and Drawbacks of [^64^Cu]Cu-DOTA-TOC

**DOI:** 10.3390/cancers14081914

**Published:** 2022-04-10

**Authors:** Nasim Vahidfar, Saeed Farzanehfar, Mehrshad Abbasi, Siroos Mirzaei, Ebrahim S. Delpassand, Farzad Abbaspour, Yalda Salehi, Hans Jürgen Biersack, Hojjat Ahmadzadehfar

**Affiliations:** 1 Department of Nuclear Medicine, Vali-Asr Hospital, Tehran University of Medical Sciences, Tehran 1419733133, Iran; n-vahidfar@sina.tums.ac.ir (N.V.); farzanehfar@tums.ac.ir (S.F.); meabbasi@tums.ac.ir (M.A.); salehi_y@sina.tums.ac.ir (Y.S.); 2 Clinic Ottakring, Institute of Nuclear Medicine with PET-Center, 1220 Vienna, Austria; siroos.mirzaei@gesundheitsverbund.at; 3 RadioMedix, Inc., Houston, TX 77041, USA; edelpassand@exceldiagnostics.com; 4 Excel Diagnostics and Nuclear Oncology Center, Houston, TX 77042, USA; 5 Division of Nuclear Medicine, Department of Medicine, The Ottawa Hospital, University of Ottawa, Ottawa, ON K1H 8L6, Canada; fabbaspour@toh.ca; 6 Department of Nuclear Medicine, University Hospital Bonn, 53127 Bonn, Germany; hans-juergen.biersack@betaklinik.de; 7 Betaklinik Bonn, 53227 Bonn, Germany; 8 Department of Nuclear Medicine, Klinikum Westfalen, 44309 Dortmund, Germany

**Keywords:** neuroendocrine, somatostatin, radiopharmaceutical, PET

## Abstract

**Simple Summary:**

One of the most incredible advances in nuclear medicine is early detection of neuroendocrine tumors, which leads to appropriate and expedient treatment pathways. Advances made with somatostatin analogue derivatives radiolabeled with Gallium-68 clarified the paths of diagnosis and treatment properly. Despite the significant improvements, widespread efforts are in progress to attain the most specific radiopharmaceutical for this purpose. In this literature review, we will provide a short overview on the role of nuclear medicine in the diagnosis of neuroendocrine tumors focusing on [^64^Cu]Cu-DOTA-TOC as a new radiopharmaceutical with promising clinical results.

**Abstract:**

Neuroendocrine tumours (NETs) arise from secondary epithelial cell lines in the gastrointestinal or respiratory system organs. The rate of development of these tumours varies from an indolent to an aggressive course, typically being initially asymptomatic. The identification of these tumours is difficult, particularly because the primary tumour is often small and undetectable by conventional anatomical imaging. Consequently, diagnosis of NETs is complicated and has been a significant challenge until recently. In the last 30 years, the advent of novel nuclear medicine diagnostic procedures has led to a substantial increase in NET detection. Great varieties of exclusive single photon emission computed tomography (SPECT) and positron emission tomography (PET) radiopharmaceuticals for detecting NETs are being applied successfully in clinical settings, including [^111^In]In-pentetreotide, [^99m^Tc]Tc-HYNIC-TOC/TATE, [^68^Ga]Ga-DOTA-TATE, and [^64^Cu]Cu-DOTA-TOC/TATE. Among these tracers for functional imaging, PET radiopharmaceuticals are clearly and substantially superior to planar or SPECT imaging radiopharmaceuticals. The main advantages include higher resolution, better sensitivity and increased lesion-to-background uptake. An advantage of diagnosis with a radiopharmaceutical is the capacity of theranostics to provide concomitant diagnosis and treatment with particulate radionuclides, such as beta and alpha emitters including Lutetium-177 (^177^Lu) and Actinium-225 (^225^Ac). Due to these unique challenges involved with diagnosing NETs, various PET tracers have been developed. This review compares the clinical characteristics of radiolabelled somatostatin analogues for NET diagnosis, focusing on the most recently FDA-approved [^64^Cu]Cu-DOTA-TATE as a state-of-the art NET-PET/CT radiopharmaceutical.

## 1. Introduction

Neuroendocrine cells are distributed widely through the human body. In particular, neuroendocrine neoplasms (NENs) can be described as epithelial neoplasms with neuroendocrine differentiation [1]. Neuroendocrine tumours (NETs) can occur in most organs of the body and share many common pathologic features. NENs frequently occur in the digestive system and the lungs and are included in the categories of gastroenteropancreatic (GEP)-NENs [2,3]. GEP-NENs are classified into two major groups: well-differentiated neuroendocrine tumours (NETs) and poorly differentiated neuroendocrine carcinomas (NECs) [2]. Very similar to these tumours are tumours with neuroectodermal origin, which arise from primitive neuroectoderm cells (PNETs) including neuroglial cells, parenchymal cells of the pineal gland, neurons, and primitive embryonal cells of the brain and retina [3,4]. One of the most important key features of NETs is overexpression of somatostatin receptors (SSTRs) [2,5].

Somatostatin is a small neuropeptide with high expression density in the brain, peripheral neurons, endocrine pancreas, and the gastrointestinal tract [6].Due to the short biological half-life of somatostatin, stable synthetic derivatives of somatostatin have been preferred for diagnosis and therapeutic purposes in clinical procedures [6]. Despite the different rate of stable somatostatin derivatives affinities’ to SSTR subtypes, octreotide and lanreotide, play an important role in the detection of and therapy for NET malignancies [7]. It has been demonstrated that in more than 85% of NETs, the 2nd, 3rd, and 5th receptor subtypes are more overexpressed out of the six known SSTRs (SSTR1-5) [8,9,10,11]. Among all of the subtypes, subtype 2 (SSTR2) plays the most significant role in the diagnosis of NETs [12]. The SSTR subtypes, which are important factors in the diagnosis of and therapy for NETs, are distributed in multiple sections of the human body, including the central nervous system, pancreas, vascular tissue, skin, prostate and cardiac myocytes [13].

Many attempts have been made to develop gamma (γ) emitter radio-labelled somatostatin derivatives as effective diagnostic tracers for NETs. For example, somatostatin-receptor scintigraphy (SRS) has been employed for three decades in many nuclear medicine imaging centres around the world [14]. NET scintigraphy of SSTRs (particularly SSTR2), primarily initiated using [^111^In]In-diethylenediamine pentaacetate (DTPA)-octreotide with SPECT/CT modality and recently [^68^Ga]Ga-(DOTA)-somatostatin derivatives with PET/CT, have been successfully used in diagnostic procedures [15,16,17,18]. Other prospective clinical studies with Copper-64 have shown better spatial resolution compared to Gallium-68 (^68^Ga) [19,20,21]. The shorter positron mean range of Copper-64 (^64^Cu) (1 mm versus 4 mm for ^68^Ga) and longer half-life (12.7 h versus 68 min for ^68^Ga) contribute to its superior imaging capabilities due to the resulting higher resolution and possible delayed imaging [22,23,24].

The concept of theranostics, which has received a great deal of attention in nuclear medicine in recent years, refers to diagnostic and therapeutic procedures conducted through a particular radio-labelled ligand with relative radionuclides for diagnosis and treatment simultaneously [25,26,27]. Theranostic pairs are those that benefit from radionuclides with appropriate physical characteristics for interchangeable diagnosis and treatment [26]. ^68^Ga, ^64^Cu, ^177^Lu and ^225^Ac are the most prevalent radionuclides for this purpose (Table 1) [26]. Interestingly, peptide receptor radionuclide therapy (PRRT) with ^177^Lu can be a theranostic agent for synchronous imaging and therapy [28,29].

In current practice, novel approaches towards specific imaging and therapeutic agents are being evaluated. Considering the importance of early-stage diagnosis and treatment of neuroendocrine-based tumours, in this review, we aimed to compare the various aspects of radiopharmaceuticals evaluated for these purposes. Agonist and antagonist receptor-based mechanisms of pharmaceuticals and the internalisation method, which occurs only with agonist based ones, directly affect diagnostic and therapeutic outcomes. These factors play very important roles, and considerable challenges have been made thus far in order to compare pharmacokinetics and choose the best possible radiopharmaceutical. We also attempt to address the significance of antagonists’ impress for imaging of NETs [29,30,31].

## 2. Somatostatin Receptor Scintigraphy with Somatostatin Analogues

In 1989, Krenning et al. introduced peptide receptor scintigraphy based on somatostatin receptor-positive tumours for the first time [32,33]. They demonstrated that [^123^I]I-204-090 can accumulate in somatostatin receptor-rich tumours and suggested that this concept can probably be applied to other receptor-based tumours [33]. [^123^I]I-Tyr^3^-octreotide has some deficiencies, including access restriction and the high expense of ^123^I, the complicated radio-labelling process, and hepatobiliary excretion, which makes the interpretation of abdominal disorders difficult [34,35]. Further studies have been conducted to address these shortcomings, through which many agents were developed, as described in Table 2.

**Figure 1:**
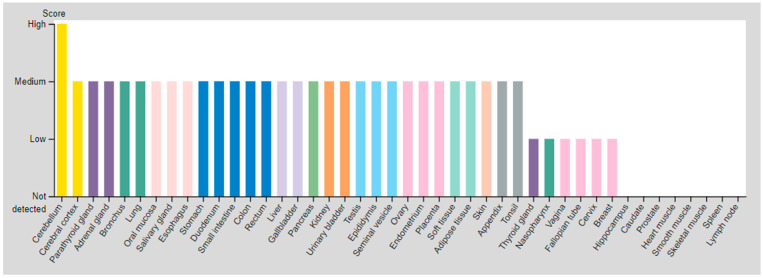

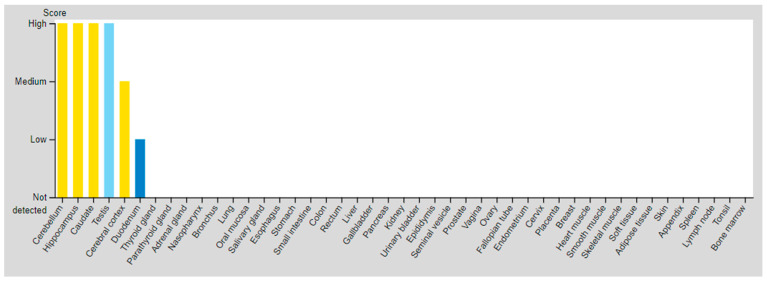
Comparison of SSTR2 (**top**) and SSTR3 (**bottom**) biodistribution based on expression rate of SSTR2,3 in mentioned tissues (From the Human Protein Atlas https://www.proteinatlas.org/ accessed on 1 April 2022.).

Reports show that octreotide derivatives have insufficient affinity to the SSTR1 and SSTR4 subtypes [13,36]. Thus, SSTR2a, 2b, 3 and 5 are the most important expressed subtypes involved in neuroendocrine-related disease [36]. SSTR5 is frequently expressed in the adult pituitary gland, heart, small intestine, adrenal gland, cerebellum and foetal hypothalamus [37,38]. There is no evidence to suggest that SSTR5 is expressed in foetal or adult kidneys, liver, pancreas, uterus, spleen, lungs, thyroid or ovaries [37,39,40]. It has been reasonably demonstrated that high expression of SSTR2 and SSTR5 is proportional with GEP-NENs and they can be used as independent predicting factors of SSTRs overexpressing for GEP-NENs patients [41]. A brief comparison of the most important SSTR (subtype 2) and the least important one (subtype 3) is shown in (Table 3) and (Figure 1).

**Table 2:** Evaluation and expression of diverse derivatives of octreotide and their probable applications for diagnosis or therapy in confronting SSTR subtypes [28,42].

Somatostatine Analogues	Abbreviation	Sequence	Radiolabeled Compounds	Indication	SST Affinity	Refs.
EDDA-HYNIC-octreotide	HYNIC-TOC	HYNIC-DPhe-Cys-Tyr-DTrp-Lys-Thr-Cys-Thr-ol	^99m^Tc	PRS	2, 3, 5	[43]
DTPA-octreotide	DTPA-OC	DTPA-DPhe-Cys-Phe-DTrp-Lys-Thr-Cys-Thr-ol	^111^In	PRRT	2, 3, 5	[6,44,45,46]
DOTA-octreotide	DOTA-OC	DOTA-DPhe-Cys-Phe-DTrp-Lys-Thr-Cys-Thr-ol	^111^In, ^90^Y	PRRT	3	[42,47,48]
DOTA-Tyr^3^-octreotide	DOTA-TOC	DOTA-DPhe-Cys-Tyr-DTrp-Lys-Thr-Cys-Thr-ol	^123^I, ^64^Cu, ^68^Ga, ^90^Y, ^177^Lu, ^225^Ac, ^213^Bi	PRRT	2, 5	[49,50,51,52]
DOTA-Tyr^3^-octreotate	DOTA-TATE	DOTA-DPhe-Cys-Tyr-DTrp-Lys-Thr-Cys-Thr-COOH	^131^I, ^64^Cu^, 68^Ga, ^177^Lu, ^90^Y, ^225^Ac, ^213^Bi	PRRT, PRS	2	[52,53,54,55]
DOTAGA-Tyr^3^-octreotate	DOTAGA-TATE	DOTAGA-DPhe-Cys-Tyr-DTrp-Lys-Thr-Cys-Thr-COOH	^68^Ga	PRS	NA	[56]
DOTAGA-octreotide	DOTAGA-TOC	DOTAGA-DPhe-Cys-Tyr-DTrp-Lys-Thr-Cys-Thr-ol	^68^Ga	PRS	NA	[56]
DOTA-Nal^3^-octreotide	DOTA-NOC	DOTA-DPhe-Cys-Nal-DTrp-Lys-Thr-Cys-Thr-ol	^111^In, ^90^Y, ^68^Ga	PRRT, PRS	2, 3, 5	[57,58]

SST: Somatostatin Receptor Subtypes. PRS: Peptide receptor scintigraphy. PRRT: Peptide receptor radionuclide therapy. NA: Not Available.

## 3. [^111^In]In-1,4,7,10-Tetraazacyclododecane-1,4,7,10-Tetraacetic Acid (DOTA)-Octreotide Derivatives

[^111^In]In-DTPA-dPhe^1^-octreotide ([^111^In]In-pentetreotide), with the trade name of OctreoScan^®^, was the first peptide receptor-based radiopharmaceutical used in clinical diagnosis [28,59]. The most significant advantage of [^111^In]In-pentetreotide compared to [^123^I]I-Tyr^3^-octreotide with a hepatobiliary excretion route is its rapid renal clearance [60,61]. [^111^In]In-pentetreotide became the gold standard for functional imaging of NETs, such as OctreoScan. Nevertheless, some limitations, including poor affinity of radiotracer to receptor, low spatial resolution, and high radiation dose to patient caused OctreoScan replacement [28]. The energy of ^111^In is relatively high, which causes suboptimum imaging resolution and high radiation exposure to patients [62]. These deficiencies are hampered by the use of ^111^In as an appropriate radionuclide for establishing other specific radiopharmaceuticals for detection of NETs. Therefore, efforts have been made to develop new tracers based on more applicable radionuclides such as ^99m^Tc, ^68^Ga, and ^64^Cu with higher affinity to somatostatin receptors and more favourable resolution and dosimetry specifications [62,63]. For instance, as the next step, [[^99m^Tc]Tc-N4^0^, Tyr^3^]-octreotate ([^99m^Tc]Tc-Demotate 1) in a clinical trial showed excellent pharmacokinetic properties, including faster accumulation in the tumor compared to Octreoscan^®^ [64]. Modification of Demotate 1, which leads to [[^99m^Tc]Tc-N4^0-1^, Asp^0^, Tyr^3^]-octreotate ([^99m^Tc]Tc-Demotate 2), in preclinical studies showed faster clearance and a better retention time in the tumor compared to [^111^In]In-DOTA-TATE [65].

## 4. [^99m^Tc]Tc-HYNIC-Octreotide Derivatives

Among several octreotide derivative candidates for radio-labelling, taking the advantage of appropriate physical characterizations of ^99m^Tc as a radionuclide [28,66], HYNIC-TOC core radiolabelled with ^99m^Tc stands out for clinical trials [28]. [^99m^Tc]Tc-HYNIC-TOC has demonstrated remarkable pharmacokinetics, including higher and faster accumulation in tumours, rapid blood clearance, one-day protocol and renal excretion, making this tracer a good alternative to OctreoScan [67,68]. [^99m^Tc]Tc-EDDA/HYNIC-TOC was authorised and approved for detection of primary and metastatic tumours of GEP-NETs under the trade name of Tektrotyd^®^ [28]. Currently, Tektrotyd is considered as a reliable and non-invasive technique for detection of NETs [69]. In a comparison between the [^18^F]Fludeoxyglucose ([^18^F]FDG) PET/CT scan and [^99m^Tc]Tc-Tektrotyd scintigraphy (TCT), a complementary value of TCT with FDG for detecting disease progression was demonstrated [70]. In a study by Gabriel M et al. [71], an overall sensitivity of 80%, a specificity of 94.4% and accuracy of 82.9% was reported in patients with GEP-NETs.

## 5. PET in Diagnosis of NET

Several studies on somatostatin analogues (SSA) radiolabeled with ^68^Ga or ^64^Cu have been accomplished in order to evaluate SSTR overexpression and detection of NETs. In an evidence-based meta-analysis, the role of PET with different radiopharmaceuticals has been reported [72]. In one of these studies conducted by Alevroudis et al., a combined approach of imaging with [^68^Ga]Ga-DOTA-TOC/TATE/NOC and [^18^F]FDG prior to therapy in NET patients was investigated [73]. Since [^68^Ga]Ga-DOTA-TOC/TATE/NOC can detect lesions with overexpression of SSTRs, and [^18^F]FDG can clarified increased glycolytic metabolism, based on various clinical based reports, it was concluded that this dual-functional imaging can be proposed as an appropriate predictive tool prior to PRRT [73]. In another meta-analysis of imaging studies, [^68^Ga]Ga-DOTA-TOC/TATE/NOC was found to be a crucial and decisive diagnostic and predictive procedure prior to therapy to determine who can benefit from the PRRT [74,75,76,77,78]. In a comparison meta-analysis study between [^18^F]F-DOPA and [^68^Ga]Ga-DOTA-peptides, it was concluded that both radiopharmaceuticals are accurate diagnostic tools in intestinal NETs based on achieved sensitivity [79]. However, [^18^F]F-DOPA was proposed as a first-line molecular imaging procedure in terms of better lesion detection compared to [^68^Ga]Ga-DOTA-peptides [79]. Though further multi-central large population studies are needed to confirm this approach [79].

### 5.1. [^68^Ga]Ga-DOTA-Octreotide Derivatives

Based on the higher spatial resolution of PET compared to scintigraphy (3–6 mm versus 10–15 mm), more information can be obtained for better interpretation and detection of smaller lesions [80]. The most noticeable advantages are higher sensitivity and specificity, lower radiation exposure, and faster examination time [81,82]. Among the derivatives mentioned above, radio-labelled DOTA-TOC, which is capable of being labelled with various trifold radionuclides, has impressive affinity to SSTR2, which is abundantly overexpressed in NETs [7,82,83].

The first clinical trial of [^68^Ga]Ga-DOTA-TOC was reported by Hoffman et al. [84]. They concluded that [^68^Ga]Ga-DOTA-TOC has a high detection rate because high tumour/non-target accumulation occurs during the 30 to 40 min post injection [84]. Other important specifications of [^68^Ga]Ga-DOTA-TOC include rapid renal clearance and low accumulation in the kidneys as well as the diagnostic potential of small lesions and small organs with expression of SSTRs [84]. In clinical experiments, the superiority of [^68^Ga]Ga-DOTA-TOC over OctreoScan has been demonstrated [8,82,84]. Moreover, it has been demonstrated that [^68^Ga]Ga-DOTA-TOC is applicable for accurate staging and restaging and even for detection and localisation of unknown primary NETs [82].

**Figure 2:**
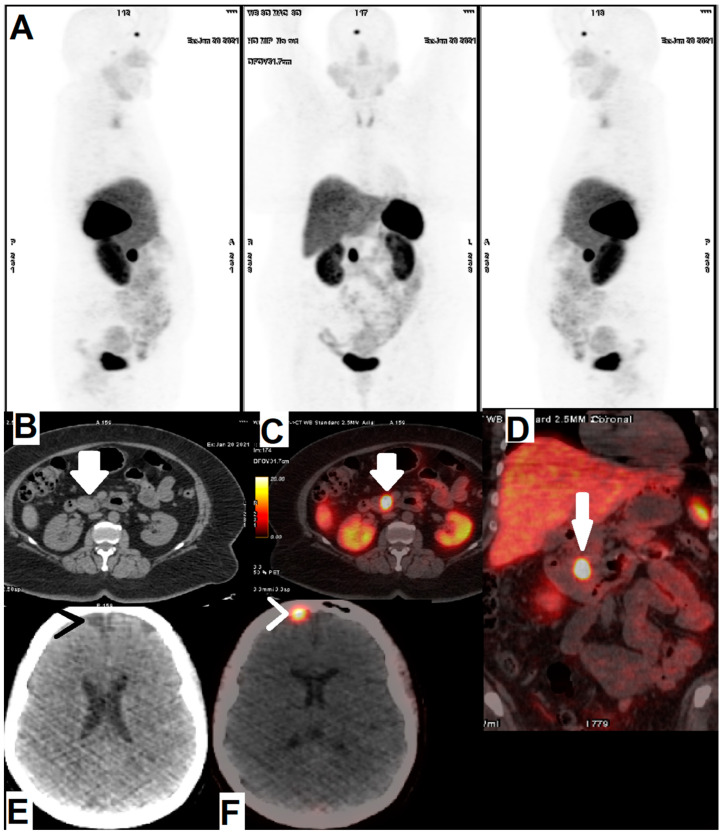
A 58-year-old man with a history of intractable peptic ulcers and hypergastrinemia referred for [^68^Ga]Ga-DOTA-TATE PET/CT scan. (**A**) MIP images in right lateral, anterior and left lateral views. Axial CT scan (**B**) and fused axial (**C**) and fused coronal (**D**) PET/CT showed a somatostatin receptor avid lesion in the pancreatic head (arrows), suggesting a NET (gastrinoma). An interesting incidental finding of a hypodense brain lesion in the right frontal lobe paramidline adjacent to falx cerebri at CT scan (**E**) showed [^68^Ga]Ga-DOTA-TATE avidity (arrow heads) on fused PET/CT (**F**) images in favor of a meningioma.

**Figure 3:**
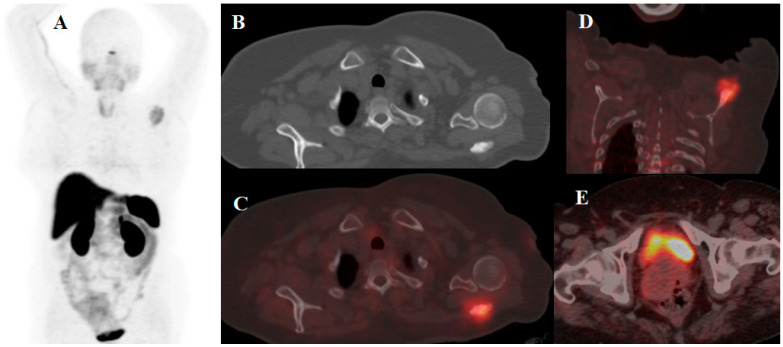
A 49-year-old woman with a history of NET of the cervix treated with local external beam radiotherapy referred for [^68^Ga]Ga-DOTA-TATE PET/CT scan due to left shoulder pain. (**A**) MIP image shows avid lesion in the in left shoulder area. Axial CT scan (**B**) and fused axial and (**C**) coronal (**D**) PET/CT images showed a sclerotic somatostatin receptor avid lesion in the body of the scapula, suggesting a NET metastasis. Interestingly, the primary tumor is in remission after local treatment (**E**).

Several clinical studies have demonstrated that [^68^Ga]Ga-DOTA-TATE is a promising tracer for diagnosis of NETs, and has more sensitivity for detection of NETs compared to OctreoScan and Tektrotyd [63,80,85,86]. [^68^Ga]Ga-DOTA-TATE is the tracer typically used in our department (Department of Nuclear Medicine, Vali-Asr Hospital, Tehran University of Medical Sciences, Tehran, Iran), and it has a good target to background and diagnostic performance (Figure 2 and Figure 3). In the clinical study by Poeppel et al., it was revealed that [^68^Ga]Ga-DOTA-TOC and [^68^Ga]Ga-DOTA-TATE have relatively equivalent detection rates in GEP-NETs [87]. Despite the high affinity of [^68^Ga]Ga-DOTA-TATE to SSTR2 (ten-fold), [^68^Ga]Ga-DOTA-TOC provided more potent diagnostic results [87,88]. Further, it was demonstrated that there is no correlation between optimal diagnosis and affinity between the peptide and receptor because accumulation of [^68^Ga]Ga-DOTA-TOC was significantly more than [^68^Ga]Ga-DOTA-TATE in NETs [87,88].

### 5.2. [^64^Cu]Cu-DOTA-Octreotide Derivatives

Several factors point to the benefit of ^64^Cu over ^68^Ga [89,90,91]. Lower positron energy (0.65MeV for ^64^Cu versus 1.90MeV for Gallium-68), which leads to a lower positron mean range (0.56mm for ^64^Cu versus 3.5mm for ^68^Ga), appears to be the most effective factor for achieving improved spatial resolution and anticipated higher diagnostic quality [92]. Recently introduced [^64^Cu]Cu-DOTA-TOC and [^64^Cu]Cu-DOTA-TATE have demonstrated preferred diagnostic imaging over [^111^In]In-DOTA-octreotide as well as [^68^Ga]Ga-DOTA-TOC for NETs [5,20,22]. The physical characteristics of ^64^Cu, including its longer half-life (12.7 h versus 1.1 h for ^68^Ga), increases the shelf life of [^64^Cu]Cu-DOTA-TOC/TATE, eliminates the necessity of a generator system and provides a flexible scanning window, which are very noticeable parameters [20,93]. Higher accumulation in tumours may be achieved in delayed images, resulting in longer ligand–receptor interaction, which is feasible with the longer half-life of ^64^Cu (Figure 1).

**Figure 4:**
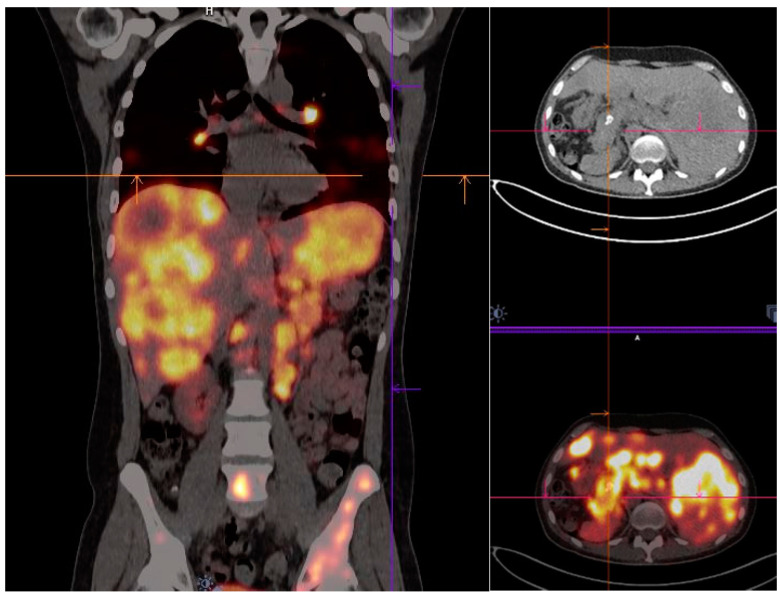
[^64^Cu]Cu-DOTA-TOC PET/CT: A 36-year-old gentleman with NET of the pancreas with Ki67 at 10%; bilobar multi-segmental multiple hepatic and multiple vertebral and pelvic bone metastases are evident in the provided coronal (**left side**) and trans-axial images (**right side**), including the liver and skeletal system.

**Figure 5:**
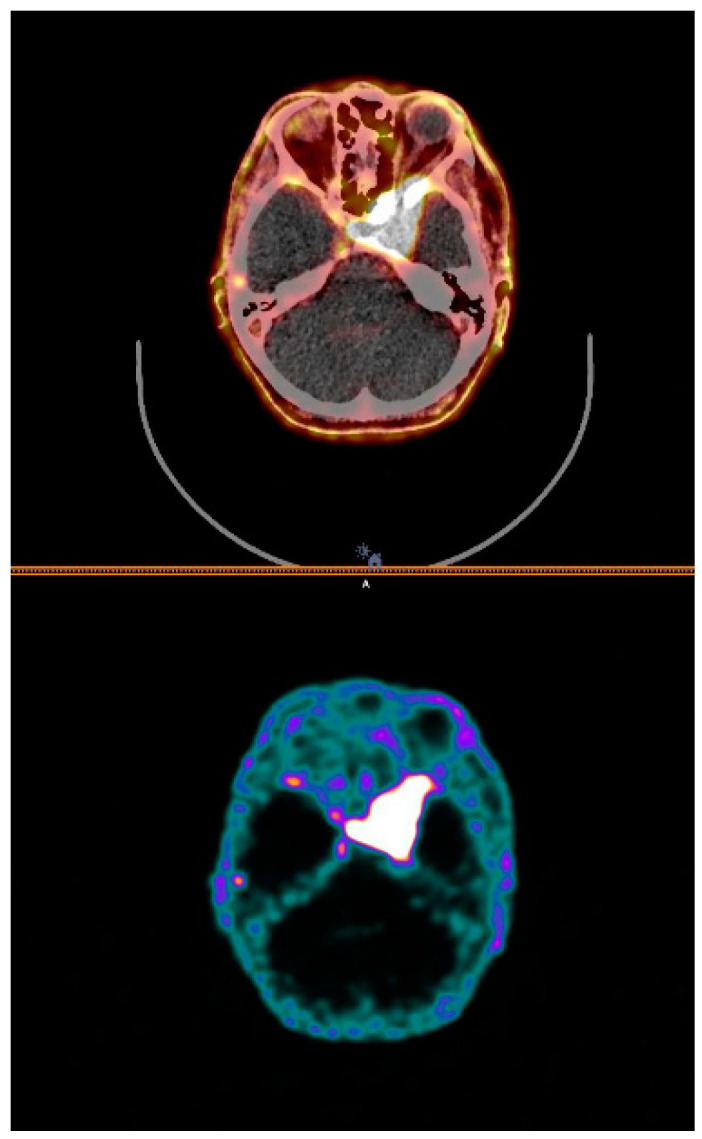
**[**^64^Cu]Cu-DOTA-TOC PET/CT: A 44-year-old gentlewoman with recurrent meningioma of the skull base; fused image (**top row**) and PET image (**bottom row**) illustrates left sphenoidal body and ala tumor involvement.

**Figure 6:**
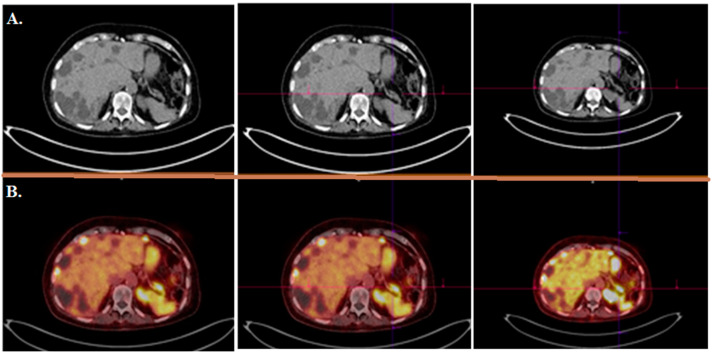
[^64^Cu]Cu-DOTA-TOC PET-CT: A 76-year-old gentlewoman with a history of resected neuro endocrine tumor of the appendix 10 years ago, with recurrence at multiple bilobar multisegmental liver metastases (image **B**); the primary tumor was detected as a suspicious lesion in the tail of the atrophic pancreas (image **A**); Ki67 9%.

The safe application of [^64^Cu]Cu-DOTA-TOC in NET patients (Figure 3, Figure 4, Figure 5 and Figure 6) as well as meningioma patients (Figure 4) is indicated by the high ratio of target to background [22,94]. The correlation of tumour detection with [^64^Cu]Cu-DOTA-TOC diagnostic images and [^177^Lu]Lu-somatostatin-derivative post-therapy images has been confirmed, demonstrating strong evidence for the validation of [^64^Cu]Cu-DOTA-TOC [22].

In a clinical trial published by Johnbeck et al., it was demonstrated that, despite the equal sensitivity of [^68^Ga]Ga-DOTA-TOC and [^64^Cu]Cu-DOTA-TATE, the latter has higher recognition potency in NET patients [20]. Based on a clinical comparison study in patients with NET reported by Malmberg et al. [23], [^64^Cu]Cu-DOTA-TATE was suggested as a feasible radiotracer for the assessment of atherosclerosis, even in the subclinical stages. The potential reason is that [^64^Cu]Cu-DOTA-TATE has higher vascular accumulation compared to [^68^Ga]Ga-DOTA-TOC [23]. More evaluations in NENs confirmed that [^64^Cu]Cu-DOTA-TATE PET/CT is particularly useful for the detection of NENs [95]. Investigations of 35 patients with NENs demonstrated that in one to three hours after injection, lesions can be detected completely [95]. With regard to these results, [^64^Cu]Cu-DOTA-TATE PET/CT could be a convenient and flexible tracer for NEN clinical evaluations [95]. The summery of clinical evaluations of [^64^Cu]Cu-DOTA-TATE and [^64^Cu]Cu-DOTA-TOC is reported in Table 4.

## 6. Antagonists versus Agonists

Pharmacomodulation is the practice of modifying existing pharmaceuticals for the purpose of improving in vivo effectiveness and drug–receptor interactions [28,96]. From a pharmacokinetic point of view, it appears that agonists are more effective than antagonists for therapeutic purposes because they can be internalised in tumour cells and the radiation doses can be elevated, which destroys tumour cells [28,97]. Recently, improved pharmacokinetics of radiopharmaceutical antagonists through the faster clearance and low renal retention was demonstrated. In addition, it was demonstrated that they have higher tumour accumulation compared to agonist-based radiopharmaceuticals [97]. Higher tumour accumulation of antagonist-based compared to agonist-based radiopharmaceuticals may be due to receptor interaction sites that are detected by antagonists. All of these factors can lead to more radiation exposure of tumour cells compared to agonists [97,98,99].

However, this theory is controversial and requires further investigation to validate it. The first trials to evaluate how antagonists act compared to agonists were conducted by Reubi et al. [100,101]. The feasibility of diagnosis of somatostatin receptors by antagonists in the clinic was proved in 2011 by discovering [^111^In]In-DOTA-BASS [30]. In this clinical trial, it was demonstrated that radiolabeled antagonists are completely feasible for imaging of NETs and they can affect the effectiveness of peptide–receptor-mediated imaging and therapy [30]. Several other studies demonstrated the superiority of antagonists over agonists in the diagnosis process performed by receptor binding radiopharmaceuticals [30,100,102,103]. Based on the comparison of the diagnostic efficacy of OctreoScan^®^ and [^111^In]In-DOTA-BASS, the latter was accepted as a proof of concept for antagonist SSTR imaging [28]. An important issue was the very modest affinity of ^111^In-DOTA-BASS for SSTR2 [103]. For this reason, many investigations were conducted to overcome this drawback.

Ultimately, the second generation of antagonist compounds was developed with the structural base of DOTA-JR11 [100,104]. The high affinity of DOTA-JR11 for SSTR2 suggested the possible use of radio-labelled configurations for diagnostic and therapeutic applications [105]. In investigations of antagonist-based imaging radiopharmaceuticals, it emerged that the role of chelators as well as radionuclides may have a significant effect on the diagnosis process and in vivo pharmacokinetics. Based on this result, many modifications took place in the development of SSTR imaging radiopharmaceuticals by antagonistic receptor interaction mechanisms [97].

It appears that ^68^Ga and ^64^Cu radio-labelled derivatives have more favourable pharmacokinetics including high affinity for SSTRs, rapid clearance, and high target to non-target ratio [97]. In total, a comparison of radionuclides and chelators’ effectiveness led to the following conclusions. First, it was demonstrated that [^68^Ga]Ga-DOTA antagonist derivatives have a much lower affinity than agonists [106]. Consequently, ^64^Cu would be more beneficial, especially when the accumulation ratio in the target to non-target is considered, which is elevated during the longer half-life of ^64^Cu [28]. The study conducted by Fani et al. examined the outcome of handling in vivo procedures with ^68^Ga and ^64^Cu along with DOTA and NOTAGA chelators on three antagonist families, including LM3, JR10 and JR11 [106]. It was concluded that [^68^Ga]Ga-NODAGA-JR11 should be replaced by [^68^Ga]Ga-DOTATATE because the former had a strikingly higher accumulation rate [97,106]. Thus, ^64^Cu and ^68^Ga radio-labelled NODAGA-LM3 are promising candidates for clinical applications. In particular, ^64^Cu radio-labelled derivatives are very promising, based on the physical characteristics of ^64^Cu and ligand attributes [97].

## 7. Conclusions and Future Research Recommendations

Despite the many improvements in the diagnosis of and therapy for NETs with somatostatin derivatives, there are still unsolved problems. While DOTA-TATE derivatives have ten-fold affinity to SSTR2 compared to DOTA-TOC derivatives, it has been demonstrated that the latter has more diagnostic potency. Therefore, it appears that the affinity may not play an important role as once believed in interactions between radiolabelled somatostatin derivatives and the receptors. Another issue that is important to consider is the replacement of antagonists with agonists in order to improve diagnostic procedures. As mentioned previously, nowadays it is believed that antagonists are more appropriate for designing diagnostic radiopharmaceuticals since antagonists do not internalize into cells and they have their specific receptors in cell membranes. Furthermore, it was recently found that switching from agonists to antagonists leads to higher efficacy. Improved pharmacokinetics and binding profile are the strengths of these replacements.

Finally, the type of radionuclide used for radio-labelling should be considered. Compared to all varieties of available radio-labelled SST analogues, strong evidence suggests that ^64^Cu radio-labelled SST analogues can be safely applied for NET diagnosis. The excellent performance of [^64^Cu]Cu-DOTATATE/-DOTATOC PET/CT and accessibility of imaging during one to three hours confirmed this tracer as reproducible, practical and highly accurate for the detection of metastases from localised lesions as well as its superiority over previous radio-labelled SSTs. All of these benefits make radio-labelled [^64^Cu]Cu-SSTs a user-friendly and promising agent for future investigations.

## Figures and Tables

**Table 1:** Physical characterization of common radionuclides with clinical applications for diagnostic or therapeutic purpose of NET.

Radionuclide/Physical Properties	Half-Life (t_1/2_)	Decay Mode	Energy (Kev)	Source	Application
^68^Ga	67.71 min	EC (10.49%) β^+^ (89.14%)	1899 822	Generator/Cyclotron	Imaging
^64^Cu	12.7 h	β^+^ (19%) γ (43%) β^-^ (38.4%)	657 511–1346 573	Reactor	Imaging/Therapy
^225^Ac	9.9 days	Pure α	5935.1	Cyclotron	Therapy
^177^Lu	6.7 days	β^-^ (82.6%) γ (17.4%)	497–384–176 208–113	Reactor	Therapy/Imaging

**Table 3:** SSTR subtypes expression score (From the Human Protein Atlas https://www.proteinatlas.org/ (accessed on 1 April 2022)). General comparison of expression origins between the most important SSTR (subtype 2) and the less important one (subtype 3) in NET investigation.

SSTR	Critical Organs’ Tissue (High Expressed)	Critical Organs’ Tissue (Medium Expressed)		Critical Organs’ Tissue (Low Expressed)
SSTR2	Cerebellum	Parathyroid gland Adrenal gland Bronchus Lung Oral mucosa Salivary gland Esophagus Stomach Duodenum Small intestine Colon Rectum Liver Gallbladder Pancreas Kidney Urinary bladder Testis	Epididymis Seminal vesicle Prostate Ovary Endometrium Placenta Adipose tissue Peripheral nerve Fibroblasts Keratinocytes Langerhans Melanocytes Epidermal cells Glandular cells Squamous epidermal cells	Cerebral cortex Thyroid gland Nasopharynx Vagina Fallopian tube Cervix, uterine Breast
SSTR3	Testis (Pachytene spermatocytes/Round or early spermatids)	Cerebral cortex Hippocampal formation Basal ganglia Cerebellum Testis (Peritubular cells)		Duodenum Testis (Spermatogonia cells)

**Table 4:** Clinical assessments of [^64^Cu]Cu-DOTA-TATE and [^64^Cu]Cu-DOTA-TOC reported in recent years.

[^64^Cu]Cu-DOTA-Octreotide Derivatives	Disease	Patients Included in the Study	Year	Result	Refs.
[^64^Cu]Cu-DOTA-TATE	NET	12 patients divided into 3 dose groups	2020	This protocol was introduced as a safe imaging method provides high quality and accurate images using optimal dose of 148 MBq (4.0 mCi) injection	[92]
	NET	60	2015	Potential role of ^64^Cu-DOTATATE in the assessment of atherosclerosis was confirmed	[23]
	NEN	128	2020	The study demonstrated prediction potency of [^64^Cu]Cu-DOTA-TATE in PFS	[24]
	NET	112	2015	Superiority of [^64^Cu]Cu-DOTA-TATE over [^111^In]In-DTPA-OC was proved	[5]
	NEN	35	2020	Excellent performance of [^64^Cu]Cu-DOTA-TATE PET/CT during 1–3 h after injection was clarified	[95]
[64Cu]Cu-DOTA-TOC	NET	33	2019	High detection rate and high target to background ratio in images raised [^64^Cu]Cu- DOTA-TATE as a promising and safe radiolabeled SST derivative for NET detection	[22]

PFS: progression-free survival. SST: Somatostatin Receptor Subtypes.
